# Supplementary material for: Direct Photochemical Synthesis of Substituted Benzo[b]fluorenes
Source: Org Lett. 2024 Nov 25;26(48):10364–8. doi: 10.1021/acs.orglett.4c03978 (PMC11629382; doi:10.1021/acs.orglett.4c03978)
Supplement: Supplementary file 2 — ol4c03978_si_002.zip [file ol4c03978_si_002.zip › FID for Publication/5r/20240726_RC-2-C-5-133A_01/plots/RC-2-C-5-133A_gHMBCAD_20240726_01_plot01.pdf]

RC-2-C-5-133A

Location	**29**	Pulse sequence	**gHMBCAD**	Temperature	**25**	Suboperator	**RuairiCrawford**
Date collected	**2024-07-26**	Solvent	**acetone**	Spectrometer	**localhost.localdomain-vnmrs500**	Operator	**MBaumann**

SAMPLE

date	**Jul 26 2024**
solvent	**acetone**
sample	**20240726_RC-2-C-5-133A_01**

ACQUISITION

sw	**4496.4**
at	**0.150**
np	**1348**
fb	**4000**
ss	**32**
d1	**1.000**
nt	**8**

2D ACQUISITION

sw1	**30154.5**
ni	**256**
phase	**arrayed**

PRESATURATION

satmode	**n**
wet	**n**

TRANSMITTER

tn	**H1**
sfrq	**499.724**
tof	**-234.7**
tpwr	**58**
pw	**6.300**

DECOUPLER

dn	**C13**
dof	**1285.1**
dm	**nnn**
decwave	**W40_P7374**
dmf	**32258**
dpwr	**43**
pwxlvl	**62**
pwx	**14.000**

HMBC

j1xh	**146.0**
jnxh	**8.0**

ADIABATCIC

pwx180ad	**P7374_ad300**
pwxlvl180	**57**
pwx180	**465.4**

FLAGS

hs	**nn**
sspul	**y**
PFGflg	**y**
hsglvl	**5736**

SPECIAL

temp	**25.0**
gain	**36**
spin	**0**

GRADIENTS

gzlvl1	**478**
gt1	**0.001000**
gzlvl3	**1434**
gt3	**0.001000**
gstab	**0.000500**

F2 PROCESSING

sb	**-0.075**
sbs	**not used**
fn	**2048**

F1 PROCESSING

gf1	**0.008**
gfs1	**not used**
proc1	**lp**
fn1	**2048**

DISPLAY

sp	**15.8**
wp	**4496.4**
sp1	**-1883.5**
wp1	**30154.5**
rfl	**-15.8**
rfp	**0**
rfl1	**1883.5**
rfp1	**0**

PLOT

wc	**142.6**
sc	**0**
wc2	**127.1**
sc2	**12.7**
vs	**141**
th	**2**
ai	cdc av

ACQUISITION ARRAYS

array	**phase**
arraydim	**512**
i	phase
1	**1**
2	**2**

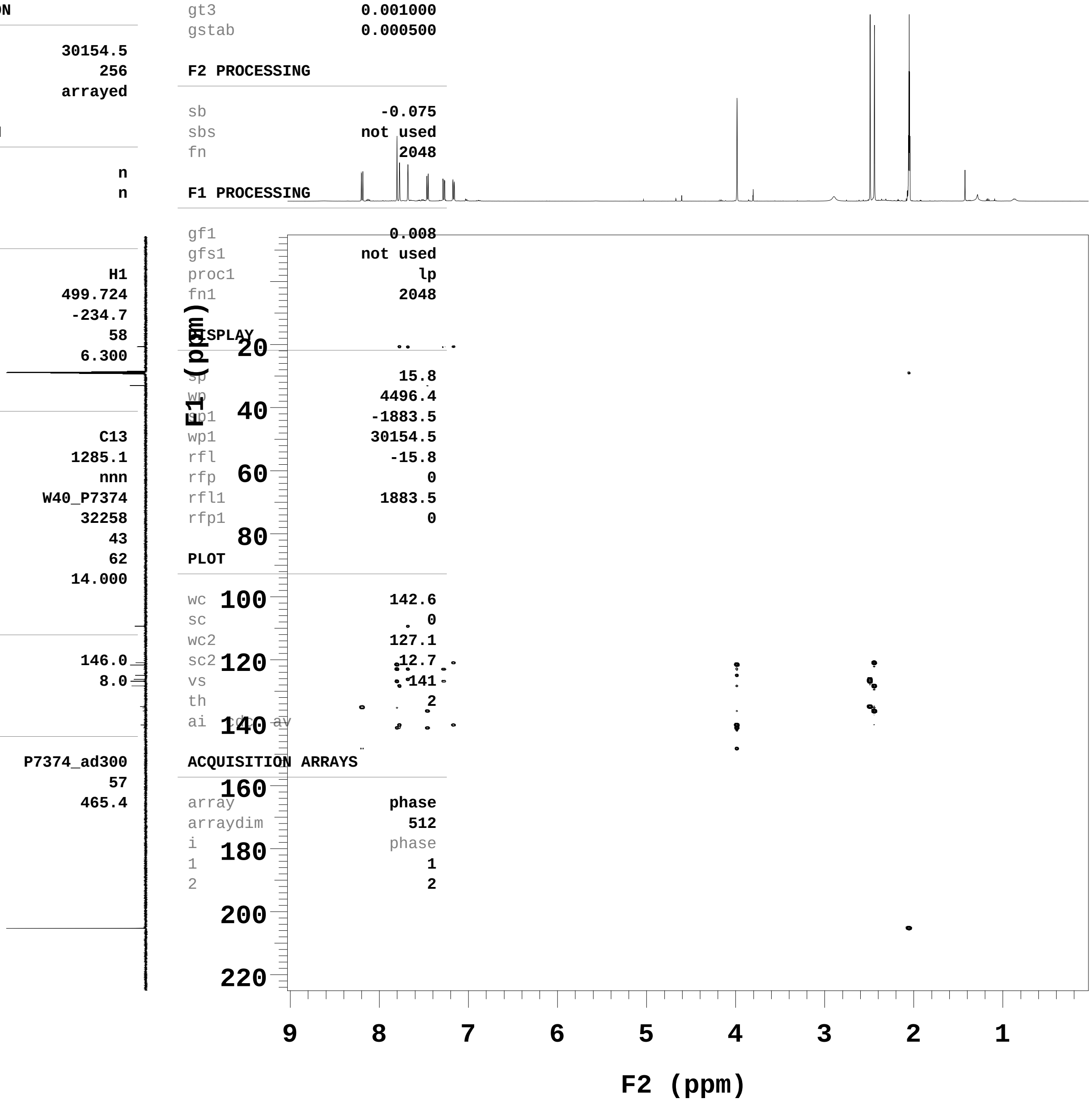

Plotname: RC-2-C-5-133A_gHMBCAD_20240726_01_plot01

Data file /home/data/MBaumann/cscb500/RuairiCrawford/2024/20240726_RC-2-C-5-133A_01/RC-2-C-5-133A_gHMBCAD_20240726_01.fid Plot date 2024-07-26
